# Dichotic sound localization properties of duration-tuned neurons in the inferior colliculus of the big brown bat

**DOI:** 10.3389/fphys.2014.00215

**Published:** 2014-06-10

**Authors:** Riziq Sayegh, Brandon Aubie, Paul A. Faure

**Affiliations:** McMaster Batlab, Department of Psychology, Neuroscience & Behaviour, McMaster UniversityHamilton, ON, Canada

**Keywords:** auditory neurophysiology, binaural hearing, dichotic stimulation, *Eptesicus fuscus*, sound localization

## Abstract

Electrophysiological studies on duration-tuned neurons (DTNs) from the mammalian auditory midbrain have typically evoked spiking responses from these cells using monaural or free-field acoustic stimulation focused on the contralateral ear, with fewer studies devoted to examining the electrophysiological properties of duration tuning using binaural stimulation. Because the inferior colliculus (IC) receives convergent inputs from lower brainstem auditory nuclei that process sounds from each ear, many midbrain neurons have responses shaped by binaural interactions and are selective to binaural cues important for sound localization. In this study, we used dichotic stimulation to vary interaural level difference (ILD) and interaural time difference (ITD) acoustic cues and explore the binaural interactions and response properties of DTNs and non-DTNs from the IC of the big brown bat (*Eptesicus fuscus*). Our results reveal that both DTNs and non-DTNs can have responses selective to binaural stimulation, with a majority of IC neurons showing some type of ILD selectivity, fewer cells showing ITD selectivity, and a number of neurons showing both ILD and ITD selectivity. This study provides the first demonstration that the temporally selective responses of DTNs from the vertebrate auditory midbrain can be selective to binaural cues used for sound localization in addition to having spiking responses that are selective for stimulus frequency, amplitude, and duration.

## 1. Introduction

Neurons throughout the auditory pathway respond selectively to the frequency and amplitude of an auditory stimulus. There is also a class of neurons first found in the auditory midbrain that have responses selective for a third acoustic parameter—signal duration—and these cells are called duration-tuned neurons (DTNs). In mammals, DTNs were first discovered from the inferior colliculus (IC) of echolocating bats (Jen and Schlegel, 1982; Pinheiro et al., 1991; Casseday et al., 1994; Ehrlich et al., 1997; Fuzessery and Hall, 1999; Casseday et al., 2000; Faure et al., 2003; Mora and Kössl, 2004). Foraging echolocating bats emit a stereotyped sequence of sounds that systematically shorten in duration as the bat closes on its prey (Faure and Barclay, 1994; Thomas et al., 2004). The presence of DTNs in bats and the finding that neural tuning for stimulus duration generally matches echolocation vocalization duration has naturally led to the hypothesis that one function of DTNs is to encode echo duration changes while hunting (Ehrlich et al., 1997; Sayegh et al., 2011); however, DTNs have also been reported from the auditory midbrain, thalamus, and cortex of non-echolocating animals (frog: Potter, 1965; Narins and Capranica, 1980; Leary et al., 2008; mouse: Brand et al., 2000; Xia et al., 2000; rat: Pérez-González et al., 2006; chinchilla: Chen, 1998; guinea pig: He, 2002; Wang et al., 2006; cat: He et al., 1997), and from the visual cortex of cats (Duysens et al., 1996). Therefore, the role(s) of DTNs within the central nervous system (CNS) cannot be exclusive to hearing or echolocation. Although DTNs have been most extensively studied in bats, and are presumably important for echolocation, the fact that DTNs are found in more than one taxon and in more than one sensory modality demonstrates that duration selectivity is an fundamental aspect of sensory processing in the vertebrate CNS. For a recent review on duration tuning in both echolocating and non-echolocating vertebrates, see Sayegh et al. (2011).

Studies on DTNs have typically used monaural (monotic) stimulation with sounds presented to the contralateral ear (e.g., Fremouw et al., 2005; Pérez-González et al., 2006), or free-field binaural stimulation with the loudspeaker positioned in the contralateral sound field to evoke neural responses (e.g., Jen and Feng, 1999; Jen and Wu, 2006). Prior to this study, only a handful of reports had used dichotic stimulation to evaluate the binaural response properties of DTNs in the mammalian IC (e.g., Brand et al., 2000; Covey and Faure, 2005). A recent electrophysiology study used dichotic paired tone stimulation and discovered that the monaural central auditory pathways contain all of the circuitry necessary for creating duration-tuned responses in the IC of the bat (Sayegh et al., 2014). Although monaural pathways were sufficient for the creation of duration-selective responses, about half of the DTNs Sayegh et al. (2014) recorded from also received an inhibitory input evoked through stimulation of the ipsilateral ear. Although the role of ipsilateral inhibition to DTNs is unclear, one likely possibility is that it modulates the contralaterally-evoked excitation received by the cell thus shaping its interaural level difference tuning profile (Grothe et al., 2010).

Two acoustic cues important for sound localization are the interaural level difference (ILD) and interaural time difference (ITD). When sound originates from a location directly in front of a listener (i.e., at 0° azimuth along the auditory midline), the received amplitude of the signal should be equal in both ears. Similarly, the path length and propagation time required for sound energy to travel to each ear should also be equal. When a sound source moves away from 0° azimuth and to the left of the auditory midline, the stimulus reaching the left ear will be larger in amplitude and will be received at a shorter time delay compared to the signal received by the right ear. The case is opposite for a sound source located to the right of midline. Interaural amplitude and time of arrival differences for sounds received by the two ears are important acoustic cues that the CNS uses to compute sound source location. Binaural ILD and ITD cues are first processed in the superior olivary complex within the auditory brainstem. In mammals, ITD cues are first processed in the medial superior olive (Goldberg and Brown, 1969; Yin and Chan, 1990) whereas ILD cues are first processed in the lateral superior olive (Boudreau and Tsuchitani, 1968; Park et al., 1996; Tollin and Yin, 2002; Tollin, 2003). Neurons located within both auditory nuclei send projections to the IC (Zook and Casseday, 1982), and ILDs appear to be recalculated in a subset of midbrain neurons (Li et al., 2010; Pollak, 2012).

This study had two major goals. First, we wanted to determine if the spiking responses of DTNs from the IC of the big brown bat (*Eptesicus fuscus*) were selective to binaural ILD and ITD cues, thus establishing if their responses could possibly play a role in sound localization. Second, we wanted to compare the response properties and binaural selectivity of DTNs and non-DTNs to determine if these two neural subpopulations could be providing the CNS with different types of information relevant for sound localization. According to the duplex theory of sound localization, ILD cues dominate the localization of high-frequency sounds whereas ITD cues dominate the localization of low-frequency sounds (Rayleigh, 1907). Because the cochlea and central auditory system of echolocating bats are primarily tuned to ultrasonic frequencies, this predicts that midbrain neurons in the bat should favor ILD processing over ITD processing, and behavioral data on the ability of *Eptesicus fuscus* to discriminate between left and right sourced sounds supports this prediction (Koay et al., 1998). Therefore, our study focused mainly on ILD processing in the bat, although whenever possible we explored the binaural properties of DTNs and non-DTNs to both binaural cues. The results reveal that many neurons in the IC of the bat, including DTNs, have binaurally-selective responses, with the majority of DTNs and non-DTNs showing ILD selectivity and almost half showing ITD selectivity. Our study provides the first evidence that neurons selective for the frequency, amplitude, and duration of an auditory stimulus can also have responses that are selective to binaural cues important for sound localization.

## 2. Methods

### 2.1. Surgical procedures

Electrophysiological recordings were obtained from male and female *Eptesicus fuscus*. To facilitate multiple recordings and to precisely replicate the position of the head between sessions, a stainless steel post was glued to the bat's skull. Prior to the head-posting surgery, bats were given a subcutaneous injection of buprenorphine (0.03 mL; 0.025 mg/kg). For the surgery, bats were first placed in a 12 × 10 × 10 cm anaesthesia induction chamber where they inhaled a 1 to 5% isofluorane:oxygen mixture (flow rate: 1 L/min). Anesthetized bats were then placed in a foam-lined body restraint within a stereotaxic alignment system that had a custom gas mask for continuous anesthetic inhalation (David Kopf Instruments Model 1900). The hair covering the skull was shaved and the overlying skin was disinfected with Betadine® surgical scrub. Local anesthetic (0.2 mL bupivicaine; 5 mg/mL) was injected subcutaneously prior to making a midline incision in the scalp. The temporal muscles were reflected, the skull was scraped clean and swabbed with ethanol, and the headpost was glued to the skull overlying the cortex with cyanoacrylate superglue (Henkel Loctite Corporation) cured with liquid acrylic hardener (Zipkicker; Pacer Technology). One end of a chlorided silver wire that was attached to the headpost was placed under the temporal muscles and served as the reference electrode. Recordings began 1–4 days after surgery and each bat was used in 1 to 8 sessions lasting ca. 6 to 8 h. Recordings were terminated if a bat showed signs of discomfort. Between sessions, the electrode penetration site was covered with a piece of contact lens and Gelfoam coated in Polysporin®. Bats were housed individually in a temperature- and humidity-controlled room. All procedures were approved by the McMaster University Animal Research Ethics Board and were in accordance with the Canadian Council on Animal Care.

### 2.2. Electrophysiological recordings

Electrophysiological recordings were conducted inside a double-walled, sound attenuating booth with electrical shielding (Industrial Acoustics Co., Inc.). Prior to recording, each bat was given a subcutaneous injection of a neuroleptic (0.3 mL; 1:1 mixture of 0.05 mg/mL fentanyl citrate and 2.5 mg/mL droperidol; 19.1 mg/kg). Bats were then placed in a foam-lined body restraint that was suspended by springs within a small animal stereotaxic frame that was customized for bats (ASI Instruments) and mounted atop of an air vibration table (TMC Migro-g). The bat's head was immobilized by securing the headpost to a stainless steel rod attached to a manipulator (ASI Instruments) mounted on the stereotaxic frame. The dorsal surface of the IC was exposed for recording by making a small hole in the skull and dura mater with a scalpel and a sharpened wire. Single-unit extracellular recordings were made with thin-wall borosilicate glass microelectrodes with a capillary filament (o.d. = 1.2 mm; A-M Systems, Inc.) and filled with 3M NaCl. Electrode resistances typically ranged from 10 to 30 MΩ. Electrodes were positioned over the dorsal surface of the IC with manual manipulators (ASI Instruments) and were advanced into the brain with a stepping hydraulic micropositioner (Kopf Model 2650). Action potentials were recorded with a Neuroprobe amplifier (A-M Systems Model 1600) whose 10x output was bandpass filtered and further amplified (500 to 1000×) by a Tucker Davis Technologies spike pre-conditioner (TDT PC1; lowpass *f_c_* = 7 kHz; high-pass *f_c_* = 300 Hz). Spike times were logged on a computer by passing the PC1 output to a spike discriminator (TDT SD1) and an event timer (TDT ET1) synchronized to a timing generator (TDT TG6).

### 2.3. Stimulus generation and data collection

Stimulus generation and on-line data collection were controlled with custom software that displayed spike-times as dot raster displays ordered by the acoustic parameter that was varied (see Faure et al., 2003). Briefly, sound pulses (e.g., pure tones) were digitally generated with a two-channel array processor (TDT Apos II; 357 kHz sampling rate) optically interfaced to two digital-to-analog (D/A) converters (TDT DA3-2) whose individual outputs were fed to low-pass anti-aliasing filters (TDT FT6-2; *f_c_* = 120 kHz), programmable attenuators (TDT PA5) and signal mixers (TDT SM5) with equal weighting. The output of each mixer was fed to a manual attenuator (Leader LAT-45) before final amplification (Krohn-Hite Model 7500). Unless testing for binaural responses, stimuli were presented monaurally, contralateral to the IC being recorded, using a Brüel & Kjær $\frac{1}{4}$ inch condenser microphone (Type 4939; protective grid on) modified for use as a loudspeaker with a transmitting adaptor (B&K Type UA-9020) to correct for nonlinearities in the transfer function (Frederiksen, 1977). The loudspeaker was positioned ca. 1 mm in front of the external auditory meatus. The output of the speaker, measured with a B&K Type 4138 $\frac{1}{8}$ inch condenser microphone (90^o^ incidence; grid off) connected to a measuring amplifier (B&K Type 2606) and bandpass filter (K-H Model 3500), was quantified with a sound calibrator (B&K Type 4231) and expressed as decibels sound pressure level (dB SPL re 20 μPa) equivalent to the peak amplitude of continuous tones of the same frequency. The loudspeaker transfer function was flat ±6 dB from ca. 28 to 118 kHz, and there was at least 30 dB attenuation at the ear opposite the source (Ehrlich et al., 1997). For presentation of binaural stimuli, two matched Brüel & Kjær $\frac{1}{4}$ inch condenser microphones were used. All stimuli had rise/fall times of 0.4 ms shaped with a square cosine function and were presented at a rate of 3 Hz.

Single units were found by searching with short duration pure tones and/or downward frequency modulated sweeps. Upon unit isolation, we determined each cell's best excitatory frequency (BEF; kHz), acoustic spiking threshold (dB SPL), and for DTNs we also determined the best duration (BD) and duration tuning response class (i.e., shortpass, bandpass or longpass DTN; see Faure et al., 2003; Fremouw et al., 2005) by presenting cells with BEF stimuli that were randomly varied in duration between 1 and 25 ms (note: the minimum stimulus duration was 1 ms). To measure the response properties of cells not selective for stimulus duration (i.e., non-DTNs), it was first necessary to select a stimulus duration for evoking neural responses. We did this by noting stimulus durations that evoked robust spiking and then randomly selecting a signal duration, typically between 1 and 10 ms, from among those with the highest spike counts. We used this procedure so that DTNs and non-DTNs would be tested with BEF signals that were comparable in duration. For all cells, the BEF and BD were determined with stimuli at +10 dB above threshold.

### 2.4. Measuring ILD and ITD binaural response functions

Upon recording the monaural (monotic) electrophysiological response characteristics of a cell, we then used binaural (dichotic) stimulation to systematically vary ILD and ITD acoustic cues to explore and compare the response properties of DTNs and non-DTNs. For DTNs the signal duration was set to the cell's BD, whereas for non-DTNs the signal duration was set to the same duration that was used to measure the neuron's BEF (see above). To measure the ILD response function of a cell, the SPL of the BEF signal presented to the contralateral ear was held at +10 dB (re threshold) while the SPL of the BEF signal presented to the ipsilateral ear was randomly varied in 5 dB steps ranging from −30 to +30 dB (re contralateral SPL). Upon collecting these data, the SPL of the contralateral stimulus was increased to +20 dB (re threshold) and the evoked responses were recollected over the same range of ILDs. To measure the ITD response function of a cell, the SPL of the BEF signals presented to the contralateral and ipsilateral ears were equal and set to +10 dB (re contralateral threshold) while the onset time of the signal presented to the ipsilateral ear was randomly varied in 10 μs steps from −250 to 250 μs (re contra stimulus). Upon collecting these data, the SPL of the binaural stimulus was increased to +20 dB (re contra threshold) and the evoked responses were recollected over the same range of ITDs. To facilitate the direct comparison of spiking responses evoked by different cells to varying ILD/ITD cues, spike counts were normalized by dividing the observed spike count at each ILD/ITD step by the maximum spike count evoked by the cell during ILD/ITD testing.

For neurons with monotonic or near-monotonic ILD/ITD response functions, we fitted a four-parameter sigmoid function in a manner similar to Tollin et al. (2008) and Karcz et al. (2012) using the equation:

(1) $$F(x)=\left(\left(A-D)/(1+({\left(x/C\right)}^{B})\right)\right)+D,$$

where *x* is the ILD or ITD value, *A* is the minimum asymptote, *B* is the slope factor, *C* is the inflection point, and *D* is the maximum asymptote. Using the fitted curves, we measured and compared the ILD_50_ or ITD_50_ point (i.e., the stimulus value eliciting 50% of the maximum spiking response; Wise and Irvine, 1985; Park and Pollak, 1993) and slope factors between DTNs and non-DTNs.

The maximum value for the biologically relevant range of ITDs in *Eptesicus fuscus* depends on the time it takes for sound to travel from one ear to the other. We measured the interaural distance (d) in five bats and its value was 12.08 ± 0.84 mm. Maximum ITDs occur for sounds broadcast at 90° away from the auditory midline (i.e., 0° azimuth) and can be calculated with the formula:

(2) $$ITD=\frac{d}{c},$$

where *d* is the interaural distance and *c* is the speed of sound. Assuming *c* = 344 m/s and given d = 12.08 ± 0.84 mm, this results in a maximum ITD of 35.12 ± 2.45 μs.

Maximum ITD values increase if the Woodworth formula is used to calculate the time required for sound to travel around a spherical head:

(3) $$ITD=\frac{r}{c}(sin\theta +\theta ),$$

where *r* is the spherical radius, *c* is the speed of sound, and θ is the azimuthal sound source angle (Woodworth, 1938). The maximum ITD we calculated with the Woodworth equation was 45.14 ± 3.14 μs; however, we rounded our estimate to be 50 μs to account for additional time delays caused by sound traveling around the snout, pinnae, and down the external auditory canals, and for variation in the head size of *Eptesicus fuscus*. Our rounded estimate of 50 μs for the maximum ITD for *Eptesicus fuscus* is consonant with the value of 55 μs estimated by Koay et al. (1998) but was considerably smaller than the value of 75 μs estimated by Aytekin et al. (2004).

### data analysis

Unless stated otherwise, all data are reported as the mean ± standard error (SE). The proportion of DTNs and non-DTNs with ILD- and ITD-selective response functions at +10 or +20 dB above threshold were compared with a Fisher's exact test. Two-sample t-tests were used to compare basic electrophysiological response parameters (e.g., recording electrode depth, stimulus duration, BEF, acoustic threshold, ILD_50_, ITD_50_, and slope factors) between DTNs and non-DTNs tested with binaural stimuli at two suprathreshold levels. All statistical analyses were performed in SPSS or Python (SciPy) and used an experiment-wise error rate of α = 0.05.

## 3. Results

### 3.1. General response properties

We obtained single unit extracellular recordings from 56 neurons recorded from the IC of 4 male and 17 female big brown bats. Of these, 29 (52%) had responses that were selective for the duration of an acoustic stimulus, while 27 (48%) were not duration selective (i.e., non-DTNs) because their spike counts remained at or above the 50% response maximum across all stimulus durations tested (typically 1 to 25 ms).

The population of 29 DTNs was divided into three categories based on the duration filter (tuning) characteristic of the cell. Bandpass DTNs respond maximally at their best duration (BD) and have spiking responses that decline to ≤50% of the response maximum at stimulus durations both longer and shorter than BD. Shortpass DTNs are similar to bandpass neurons in that they also respond maximally at BD; however, their spiking responses only drop to ≤50% of the maximum at stimulus durations longer but not shorter than BD. Longpass DTNs do not have a BD and spike only when the stimulus duration meets or exceeds some minimum duration. Unlike typical energy integrating sensory neurons, both the number of spikes and first-spike latencies of longpass DTNs do not increase or decrease, respectively, with increasing SPLs (Brand et al., 2000; Faure et al., 2003; Pérez-González et al., 2006). Within the population of 29 DTNs, 21 were shortpass, 6 were bandpass, and 2 were longpass. Spiking by shortpass and bandpass DTNs was phasic, responding mainly to stimulus offset, while one longpass DTN had onset-evoked sustained responses and the other had a mixed response spiking to both the onset and offset of the stimulus (depending on the SPL).

We divided the population of non-DTNs into two groups based on their spiking responses. Of 27 non-DTNs, 21 exhibited phasic (transient) spiking with 19 responding to stimulus onset, 0 to stimulus offset, and 2 showing mixed spiking responding to both the onset and offset of the stimulus. The remaining 6 cells were classified as sustained responding because they had spikes evoked throughout the duration of the stimulus.

We tested for general differences between DTNs and non-DTNs with respect to the recording electrode depth, duration of the stimulus used to evoke a response (i.e., we compared the BD of DTNs *vs.* the stimulus duration of non-DTNs), BEF, and acoustic threshold. Within the population of cells tested, the mean BEF of DTNs was significantly higher than the mean BEF of non-DTNs; however, there were no significant differences in recording electrode depth, duration of the stimulus used to evoke a response, or acoustic threshold between DTNs and non-DTNs (Table 1).

**Table 1:** **Summary statistics of general response properties of DTN and non-DTNs**.

**Parameter**	**DTN (*n* = 29)**	**Non-DTN (*n* = 27)**	***t*-test**
	**Mean** ± ***SD***	**Mean** ± ***SD***	***p*-value**
Depth (μm)	1298.73 ± 315.57	1209.11 ± 360.54	0.3346
Duration (ms)	3.55 ± 7.11	5.26 ± 4.87	0.3114
Best Excitatory Frequency (kHz)	48.26 ± 13.57	40.30 ± 12.47	**0.0471**
Threshold (dB SPL)	45.38 ± 12.52	40.85 ± 18.57	0.2954

Statistically significant values are indicated in bold face type.

### 3.2. Binaural response properties of DTNs and non-DTNs

We observed three types of binaural response properties in the population of IC neurons tested. Acoustic stimulation of the ear contralateral to the IC that was being recorded caused an excitatory (E) response in every neuron. Presentation of a stimulus to the ear ipsilateral to the IC that was being recorded evoked either an excitatory (E), an inhibitory (I), or no response (O). Of 56 neurons, 36 (65.5%; 18 DTNs) were characterized as EI because they were excited by stimulation of the contralateral ear and inhibited by stimulation of the ipsilateral ear, 12 (21.8%; 6 DTNs) as EE because they were excited by stimulation of either ear, 7 (12.7%; 4 DTNs) as EO because they were excited by stimulation of the contralateral ear and neither excited nor inhibited by stimulation of the ipsilateral ear, and 1 cell (shortpass DTN) was unclassified because we did not characterize its binaural responses in this manner.

#### 3.2.1. ILD selectivity

**Figure 1:**
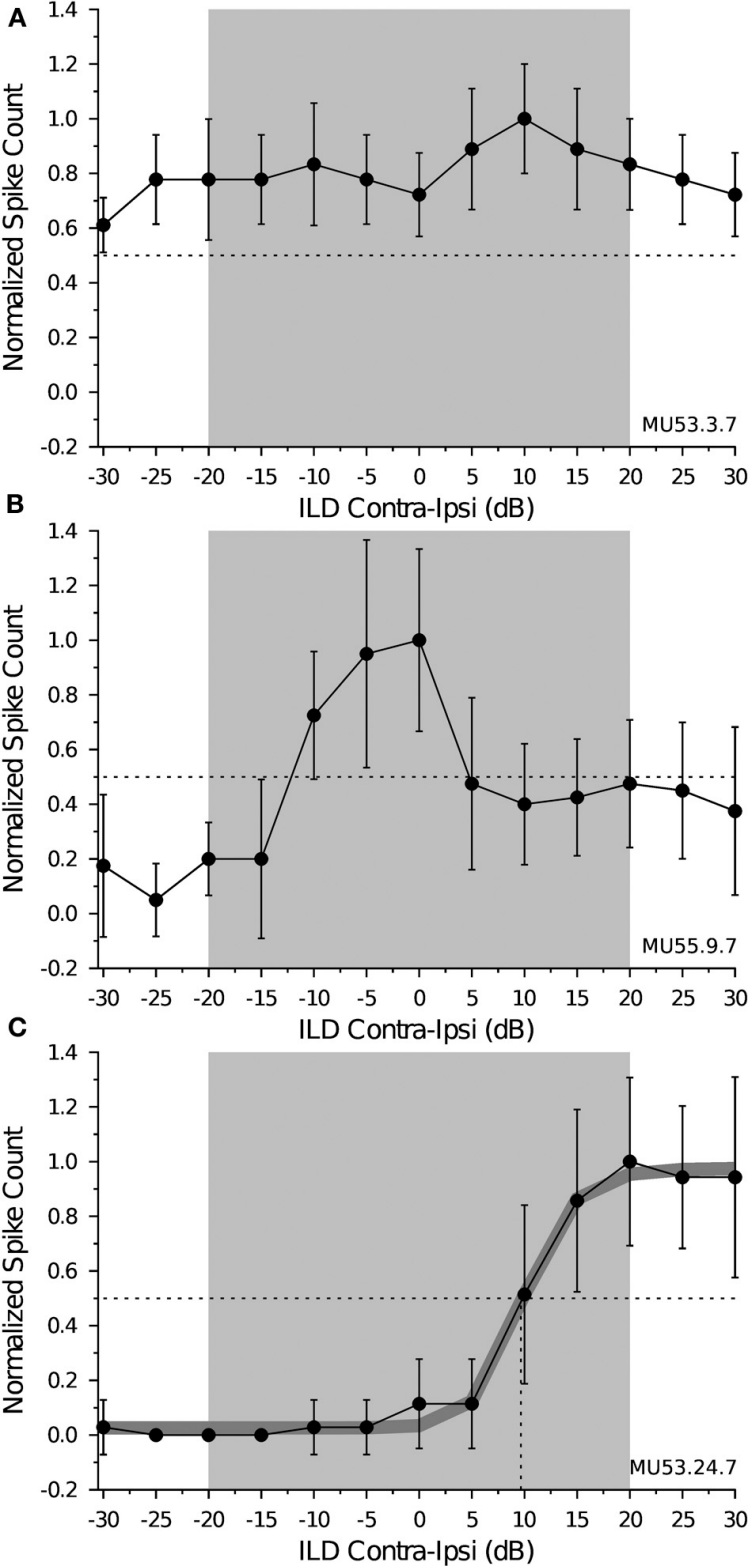
**Example interaural level difference (ILD) response functions measured from inferior colliculus (IC) neurons of *Eptesicus fuscus***. Each panel shows the mean ± SE spikes per stimulus, normalized to the response maximum and plotted as a function of binaural interaural level difference (ILD), measured in decibels (dB) as the sound pressure level (SPL) in the contralateral ear minus the SPL in the ipsilateral ear. The *gray region* spanning ± 20 dB represents a conservative estimate of the range of dominant ILD cues for an adult *Eptesicus fuscus* and the *horizontal dotted line* represents 50% of the maximum spike count. During data collection, the SPL presented to the contralateral ear was kept constant while the SPL presented to the ipsilateral ear was varied in 5 dB steps from −30 dB to +30 dB re contralateral ear. The abscissa is the binaural ILD measured as the SPL of the contralateral stimulus minus the SPL of the ipsilateral stimulus, with negative values indicating the ipsilateral signal was louder than the contralateral signal and positive values indicating the contralateral signal was louder than the ipsilateral signal. **(A)** Non-selective ILD response function. Spiking by this shortpass DTN was maintained at ≥50% of the maximum at all ILDs tested, suggesting that the responses of this cell were not directionally selective for sound azimuth. MU53.03.07: BEF = 56 kHz, BD = 1 ms, depth = 1162 μm, threshold = 27 dB SPL, level = +10 dB (re threshold), 10 trials per stimulus. **(B)** Peaked ILD-selective response function. Spiking by this shortpass DTN showed a distinct peak with marked tuning favoring sounds emanating from directly in front (ILD = 0 dB) or slightly to the ipsilateral side (ILD = −5 to −10 dB). MU55.09.07: BEF = 55 kHz, BD = 1 ms, depth = 1373 μm, threshold = 36 dB SPL, level = +10 dB (re threshold), 10 trials per stimulus. **(C)** Monotonic ILD-selective response function. This non-DTN was nearly unresponsive to the presentation of ipsilateral sounds, but then its response increased monotonically until reaching response saturation as the ILD increased and favored the contralateral ear. The *thick gray line* is the fitted 4-parameter sigmoid function, which was highly correlated with the raw data (*r* = 0.998, *p*«0.001; see Equation 1 in text). The ILD_50_ value measured from the sigmoid function was +9.66 dB (*vertical dotted line*), and the slope factor was 16.67. MU53.24.07: BEF = 41 kHz, duration = 5 ms, depth = 1384 μm, threshold = 59 dB SPL, level = +10 dB (re threshold), 10 trials per stimulus.

The ILD function of a cell shows the number of spikes evoked in response to sounds varied in their binaural SPL, defined as the stimulus level in the contralateral ear minus the stimulus level in the ipsilateral ear; negative ILD values indicate the ipsilateral signal was louder than the contralateral signal, whereas positive values indicate the contralateral signal was louder than the ipsilateral signal. We recorded ILD response functions from 54 neurons (27 DTNs, 27 non-DTNs) when the contralateral ear was held at +10 dB (re threshold) and from 47 neurons (25 DTNs, 22 non-DTNs) when the contralateral ear held at +20 dB (re threshold), and observed three types of ILD response functions across the population of cells tested (Figure 1). The first type was from cells that were non-selective for signals that were dichotically varied in ILD. These neurons were characterized by having relatively flat response functions over the entire range of binaural level differences, never dropping to ≤50% of the maximum response at any amplitude disparity. Figure 1A shows an example of a non-selective ILD response function recorded from a shortpass DTN; the 50% response point is illustrated with a *dotted line* and the *gray box* spanning from ±20 dB represents a conservative estimate of the biologically relevant range of dominant ILDs for an adult big brown bat (note: the range of relevant ILDs varies with sound frequency; see Jen and Chen, 1988 and Aytekin et al., 2004). Non-selective ILD functions were observed in 13 of 54 cells (24.1%; 5 DTNs and 8 non-DTNs) tested at +10 dB (re threshold), and 14 of 47 cells (29.8%; 4 DTNs and 10 non-DTNs) tested at +20 dB (re threshold).

The second type of ILD function we encountered was from cells with peaked responses, characterized by having a response maximum located at a specific ILD (or range of ILDs) and a function that generally dropped to ≤50% of the maximum at both positive (contralateral ear louder) and negative (ipsilateral ear louder) ILDs. Figure 1B shows an example of peaked ILD response function measured from a shortpass DTN whose responses were selective for signals with a 0 dB ILD (i.e., favoring sound sources originating along the auditory midline) or with a slightly negative ILD (i.e., favoring sound sources originating just ipsilateral to the auditory midline). Neurons with peaked ILD response functions were observed in 6 of 54 cells (11.1%; 4 DTNs and 2 non-DTNs) tested at +10 dB (re threshold), and 5 of 47 cells (10.6%; 3 DTNs and 2 non-DTNs) tested at +20 dB (re threshold).

The third and most common type of ILD function we encountered was from cells with monotonic or near-monotonic responses. Cells with monotonic ILD response functions were selective for contralateral sound sources when the function gradually increased from negative to positive ILD values within the biologically relevant range, and were selective for ipsilateral sound sources when the function gradually decreased from negative to positive ILD values within the biologically relevant range. Figure 1C shows an example of a monotonic ILD response function recorded from a non-DTN with phasic spiking. This cell was selective for sounds originating from the contralateral hemisphere because its ILD response function peaked and then saturated at the most positive ILDs within the biologically relevant range (*gray box*) demonstrating the neuron responded best when sounds were much louder in the contralateral ear.

**Figure 2:**
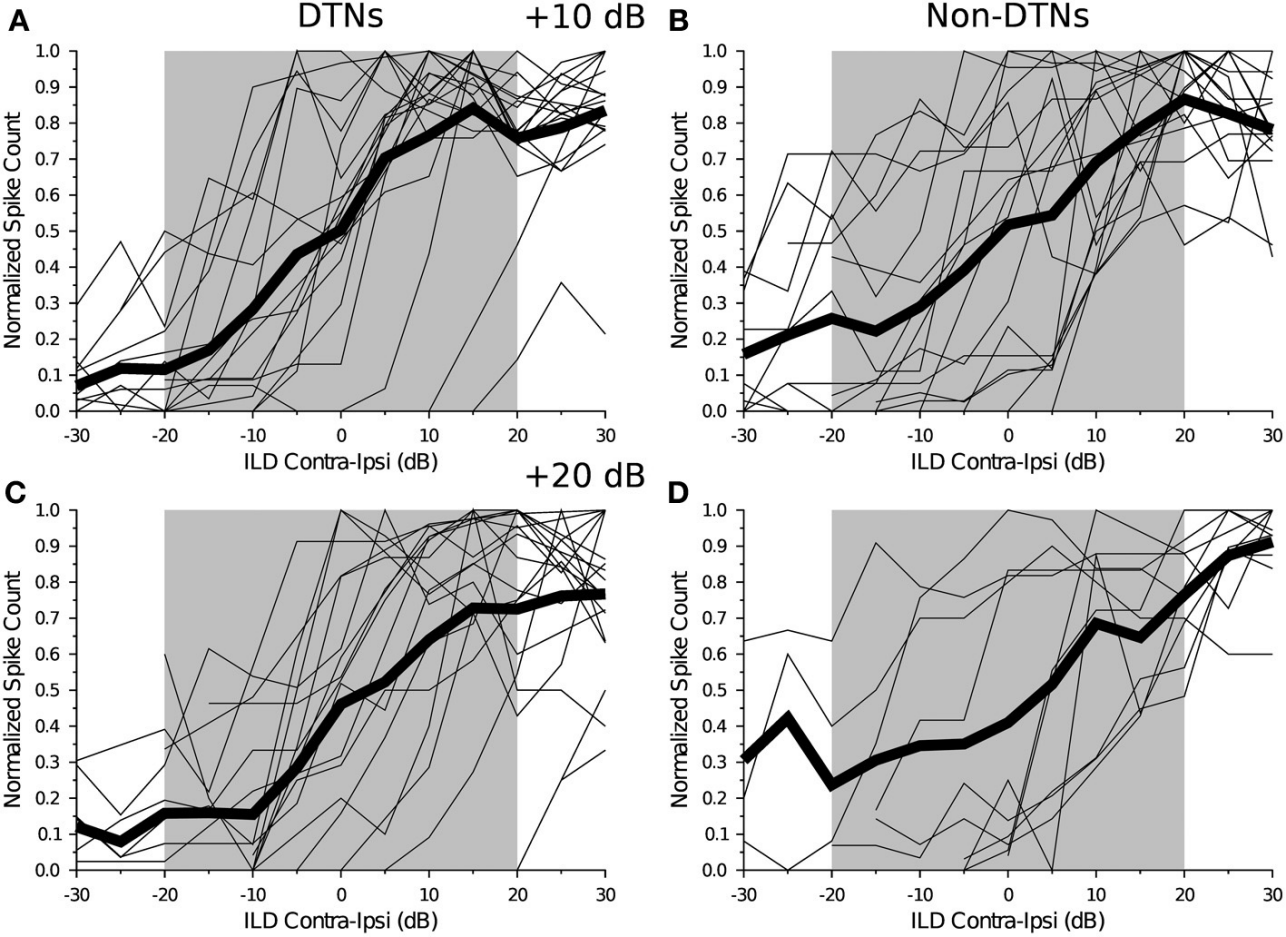
**Individual and population average monotonic ILD response functions for DTNs and non-DTNs from the IC of the big brown bat**. Each panel shows the normalized ILD response curves for individual neurons (*thin black lines*) as a function of ILD, measured in dB as the SPL in the contralateral ear minus the SPL in the ipsilateral ear, while the *bold black line* is the population average. The *gray region* is a conservative estimate of the biologically relevant range of dominant ILD cues for an adult *Eptesicus fuscus*. During data collection, the SPL presented to the contralateral ear was held constant while the SPL presented to the ipsilateral ear was varied in 5 dB steps from −30 dB to +30 dB re contralateral ear. **(A,B)** Individual monotonic ILD response functions and the population average for **(A)** DTNs and **(B)** non-DTNs measured with the contralateral ear at +10 dB (re threshold). **(C,D)** Individual monotonic ILD response curves and the population averages for **(C)** DTNs and **(D)** non-DTNs measured with the contralateral ear at +20 dB (re threshold). The shaded gray region represents a conservative estimate of the window of biological relevance (−20 to +20 ILD dB). We observed that on the population level the steepest portions of the averaged curves (both DTNs and non-DTNs) fell between the biological ILD range. **(A)** *n* = 18, **(B)** *n* = 17, **(C)** *n* = 18, **(D)** *n* = 10

Figure 2 displays all of the individual ILD curves that we measured from DTNs and non-DTNs with monotonic (or near-monotonic) ILD response functions when the level of contralateral stimulus was held at +10 dB (Figure 2A,B) and when it was held at +20 dB above threshold (Figure 2C,D). Note how the individual curves, along with their population averages, gradually increase in magnitude from negative to positive ILDs with the greatest change in the slope of the average population function centered directly over the biologically relevant range of ILDs in *Eptesicus fuscus* (Figure 2). This occurred for all cells at +10 and +20 dB above threshold, demonstrating that both DTNs and non-DTNs can have responses selective for binaural ILDs in addition to having other forms of response selectivity such as frequency tuning, amplitude tuning, and in the case of DTNs, duration tuning. Midbrain neurons with monotonic ILD response functions were observed in 35 of 54 cells (64.8%; 18 DTNs and 17 non-DTNs) tested at +10 dB above threshold, and 28 of 47 cells (59.6%; 18 DTNs and 10 non-DTNs) tested at +20 dB above threshold.

**Figure 3:**
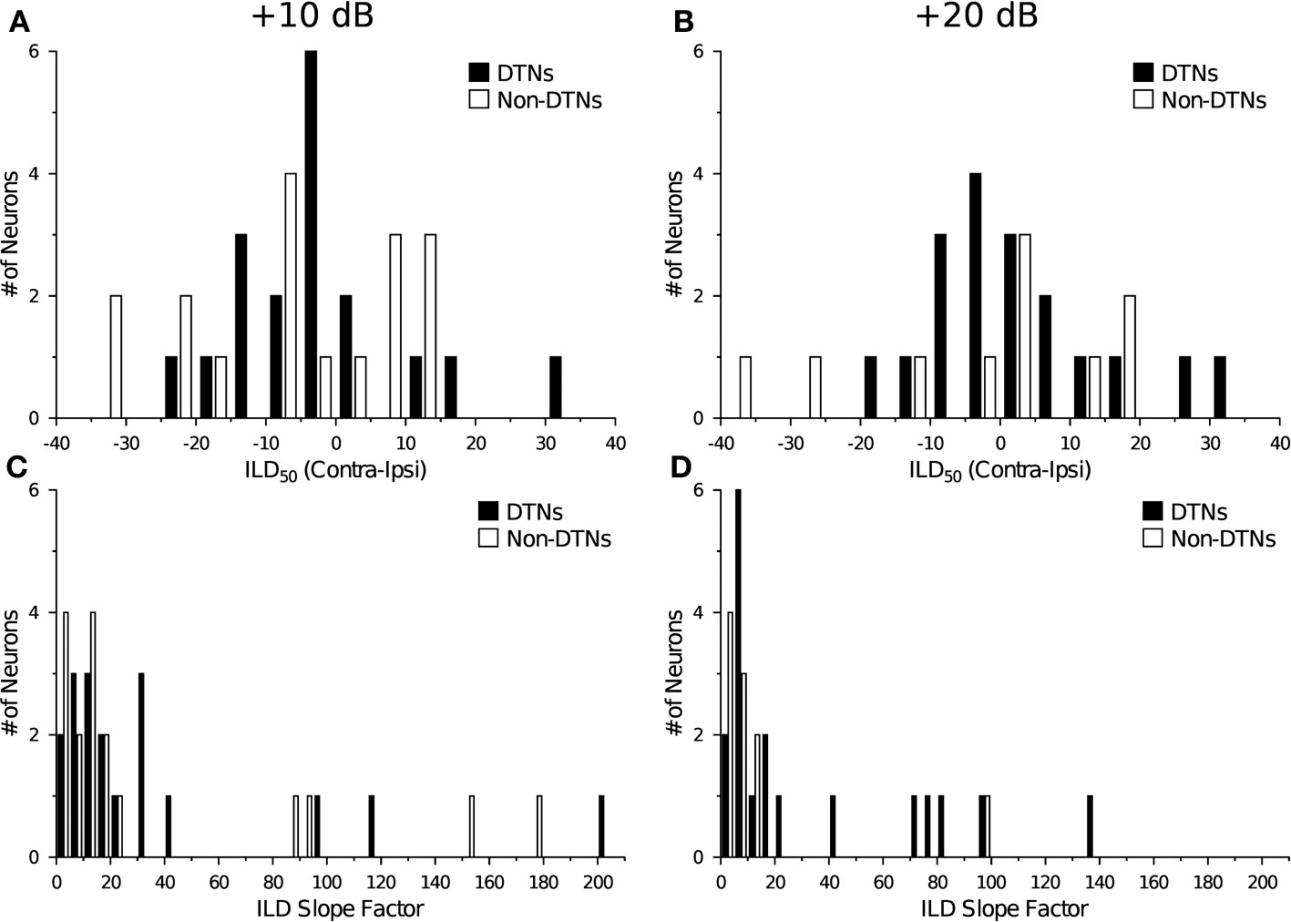
**Histograms comparing the distributions of ILD_50_ values and slope factors of DTNs and non-DTNs recorded at two suprathreshold levels**. The distributions of ILD_50_ values for DTNs and non-DTNs were not significantly different at either **(A)** +10 dB or **(B)** +20 dB (re threshold). Note that the ILD_50_ value from most neurons was centered between 0 and −10 dB, demonstrating that the midpoint of their ILD response functions was centered near the auditory midline or was slightly biased toward the ipsilateral hemifield. The distributions of ILD slope factors did not differ between DTNs and non-DTNs at either **(C)** +10 dB or **(D)** +20 dB (re threshold), with the majority of cells having slope factors ≤20.

To compare the responses and selectivity of DTNs and non-DTNs with monotonic (or near-monotonic) ILD functions, we fit a four-parameter sigmoid curve (see Equation 1) to the data from each cell and then measured the ILD value where the 50% response maximum occurred (ILD_50_; see *vertical dotted line* in Figure 1C) and calculated a slope factor for every neuron. Figure 3 shows histograms of the ILD_50_ values and slope factors measured from DTNs and non-DTNs with monotonic (or near-monotonic) ILD response functions at +10 and +20 dB above threshold. The distribution of ILD_50_ values (Figure 3A,B) and slope factors (Figure 3C,D) was similar between DTNs and non-DTNs, revealing no overt differences in ILD sensitivity between the two neural subpopulations. Closer inspection of the data in Figure 3A,B hints at the possibility of a bimodal distribution of slope factors, so we conducted a further analysis and compared the electrophysiological characteristics of neurons with slope factors <20 to those with slope factors >20 and found no differences between DTNs and non-DTNs in their recording electrode depth, stimulus duration, BEF, or acoustic threshold (data not shown).

Table 2 provides the mean ± SE and range of peak spike counts that were evoked by the stimuli we employed during ILD testing, broken down by cell type. These results indicate there were no major differences in neural responsiveness to the signals we presented for ILD testing across the different cell populations. Table 3 summarizes the type of binaural interactions that were observed for neurons with different types of ILD response selectivity.

**Table 2:** **Range and average peak spike counts (spikes per stimulus) for cells tested with ILD stimuli at +10 dB above threshold**.

	**Cell type**	***n***	**Peak spiking response**	
			**Range**	**Mean ± *SE***
DTNs	Shortpass	20	0.90-6.95	2.40 ± 0.29
	Bandpass	5	0.87-3.33	1.98 ± 0.45
	Longpass	2	3.20-6.35	4.78 ± 1.57
Non-DTNs	Phasic	21	0.87-4.67	2.18 ± 0.24
	Sustained	6	0.65-2.30	1.54 ± 0.22

**Table 3:** **Binaural interactions of IC neurons with different types of ILD response functions**.

**Amplitude**	**ILD response**	**EE**	**EI**	**EO**	**?**
+ 10 dB	Non-Selective	6 (1)	0 (0)	7 (4)	0 (0)
	Peaked	3 (3)	3 (1)	0 (0)	0 (0)
	Monotonic	2 (1)	33 (17)	0 (0)	0 (0)
+ 20 dB	Non-Selective	6 (1)	1 (0)	7 (3)	0 (0)
	Peaked	3 (3)	2 (0)	0 (0)	0 (0)
	Monotonic	2 (1)	26 (17)	0 (0)	0 (0)

Number of DTNs shown in parentheses. EE, cell excited by stimulation of contralateral and excited by stimulation of ipsilateral ear; EI, cell excited by stimulation of contralateral ear and inhibited by stimulation of ipsilateral ear; EO, cell excited by stimulation of contralateral ear and neither excited nor inhibited by stimulation of ipsilateral ear; ?, binaural inputs to cell not tested.

Table 4 compares the electrophysiological parameters of DTNs and non-DTNs with ILD-selective response functions measured at +10 and +20 dB (re threshold). For the purpose of this analysis, cells with peaked and monotonic (or near-monotonic) ILD response functions were grouped together under a single “ILD-selective” response category for comparison with “non-ILD-selective” cells. There was no difference in the proportion of DTNs and non-DTNs with ILD selective responses at +10 dB re threshold (Fisher's exact test, *p* = 0.5256), but at +20 dB re threshold there was a strong trend for a higher proportion of DTNs to show ILD selectivity (Fisher's exact test, *p* = 0.0533). We also compared the mean ± standard deviation (SD) electrophysiological response characteristics of DTNs and non-DTNs, but only for those cells with ILD selectivity at either +10 or +20 dB (re threshold), and found no differences in the dorsal-ventral recording electrode depth, BEF, or acoustic threshold. This result indicates that there were no overt topographical differences between DTNs and non-DTNs tested with ILD stimuli in our dataset. There was also no difference in the average duration of the acoustic stimulus that was used to evoke spiking in DTNs and non-DTNs with ILD-selective responses. In Table 4 it is also evident that the mean ILD_50_ value for DTNs and non-DTNs with monotonic (or near-monotonic) ILD response functions was located within the biologically relevant range of ILDs for *Eptesicus fuscus* at both suprathreshold levels. Moreover, there was no significant difference in the mean ILD_50_ value and slope factor between DTNs and non-DTNs at either level above threshold.

**Table 4:** **Comparison of electrophysiological parameters in DTNs and non-DTNs with ILD-selective response functions**.

**Amplitude**	**Parameter**	**DTN**		**Non-DTN**		***t*-test**
		**Mean ± *SD***	***n***	**Mean ± *SD***	***n***	***p*-value**
+ 10 dB	Depth (μm)	1309.32 ± 335.82	22	1297.26 ± 376.01	19	0.9163
	Duration (ms)	4.05 ± 8.06	22	5.84 ± 5.40	19	0.4262
	Frequency (kHz)	46.55 ± 14.33	22	40.53 ± 13.24	19	0.1834
	Threshold (dB SPL)	47.09 ± 12.28	22	46.05 ± 19.22	19	0.8397
	ILD_50_ (dB)	−2.33 ± 12.64	18	−5.70 ± 14.27	17	0.4775
	ILD Slope Factor	37.29 ± 50.06	18	37.92 ± 53.68	17	0.9726
+20 dB	Depth (μm)	1349.76 ± 318.46	21	1209.92 ± 323.44	12	0.2511
	Duration (ms)	4.10 ± 8.26	21	5.5 ± 4.91	12	0.6061
	Frequency (kHz)	48.24 ± 39.33	21	39.33 ± 12.92	12	0.0766
	Threshold (dB SPL)	47.24 ± 12.55	21	44.50 ± 20.69	12	0.6498
	ILD_50_ (dB)	2.20 ± 13.16	18	−2.97 ± 5.66	10	0.3889
	ILD Slope Factor	35.05 ± 40.63	18	15.03 ± 28.56	10	0.1808

#### 3.2.2. ITD selectivity

**Figure 4:**
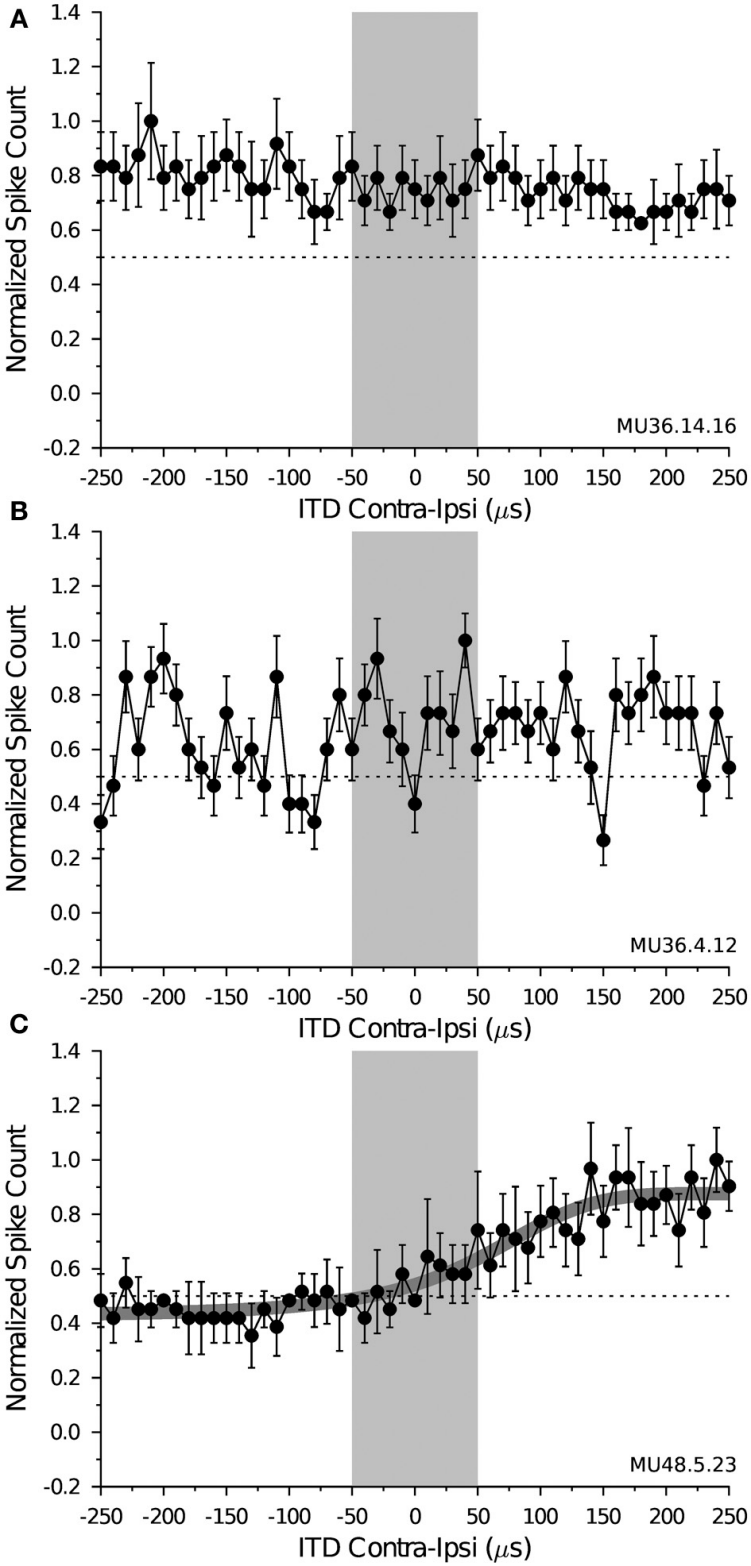
**Example interaural time difference (ITD) response functions measured from IC neurons of the big brown bat**. Each panel shows the mean ± SE spikes per stimulus, normalized to the response maximum and plotted as a function of the binaural ITD, measured as the contralateral stimulus onset time minus the ipsilateral stimulus onset time. The *gray region* spanning from ± 50 μs represents the maximum range of ITD cues for an adult *Eptesicus fuscus* and the *horizontal dotted line* represents 50% of the maximum spike count. During data collection, the SPL of the presented signal was equal in both ears while the onset time of the ipsilateral stimulus was randomly varied in 10 μs steps from −250 to 250 μs (re contra stimulus). The abscissa is the binaural ITD measured as the onset time of the contralateral stimulus minus the onset time of the ipsilateral stimulus, with negative values indicating the ipsilateral signal led the contralateral signal and positive values indicating the contralateral signal led the ipsilateral signal. **(A)** Non-selective ITD response function. Spiking by this shortpass DTN was maintained at ≥50% of the maximum at all ITDs tested and the function was relatively flat across the biological range of ITD cues available to the bat, suggesting that the responses of this cell were not directionally selective for sound azimuth. MU36.14.16: BEF = 44 kHz, BD = 2 ms, depth: 1225 μm, threshold = 24 dB SPL, level = +10 dB (re threshold), 15 trials per stimulus. **(B)** Cyclical ITD-selective response function. The magnitude of spiking by this neuron shows somewhat regular oscillations with peaks and troughs in its spiking in response to varying ITD cues. MU36.04.12: BEF = 46 kHz, duration = 6 ms, depth = 1486 μm, threshold = 63 dB SPL, level =+20 dB (re threshold), 20 trials per stimulus. **(C)** Monotonic ITD-selective response function. The number of evoked spikes by this cell increased as the stimulus in the ipsilateral ear was delayed (re contralateral stimulus). The *thick gray line* is the fitted 4-parameter sigmoid function, which was highly correlated with the raw data (*r* = 0.95, *p*«0.001; see Equation 1 in text). The ITD_50_ value measured from the sigmoid function was 198.60 μs and the slope factor was 4.0302. MU48.05.23: BEF = 59 kHz, BD = 1 ms, depth = 1290 μm, threshold = 35 dB SPL, level =+20 dB (re threshold), 15 trials per stimulus.

The ITD function of a cell shows the number of spikes that were evoked by sounds that were varied in their binaural time of arrival, defined as the onset time of the contralateral stimulus minus the onset time of the ipsilateral stimulus; negative ITD values indicate the ipsilateral signal led the contralateral signal, whereas positive values indicate the contralateral signal led the ipsilateral signal. We recorded ITD response functions from 45 neurons (23 DTNs, 22 non-DTNs) when the binaural stimulus level was equal in both ears and set to +10 dB (re contra threshold) and from 34 neurons (17 DTNs, 17 non-DTNs) when the binaural stimulus level was equal in both ears and set to +20 dB (re contra threshold), and observed three types of ITD response functions across the population of cells tested (Figure 4). The most common type of ITD response function was from cells that were non-selective to sounds dichotically varied in ITD. These cells were characterized by having relatively flat ITD response functions over the entire range of binaural time disparities. Figure 4A shows an example of a non-selective ITD response function recorded from a shortpass DTN; the 50% response point is illustrated with a *dotted line* and the *gray box* spanning from ±50 μs represents our estimate of the maximum biological range of ITD cues for an adult big brown bat (see calculation details above). Non-selective ITD functions were observed in 24 of 45 cells (53.3%; 11 DTNs and 13 non-DTNs) tested at +10 dB (re threshold), and 15 of 34 cells (45.5%; 7 DTNs and 8 non-DTNs) tested at +20 dB (re threshold).

The second type of response profile we encountered was from cells with cyclical ITD curves. Cyclical ITD response functions oscillate with clear peaks and troughs occurring within the biologically relevant range of ITDs, with the peaks tending to rise above and the troughs falling below the 50% response maximum. Figure 4B shows an example of a cyclical ITD response function recorded from a non-DTN with phasic spiking. Neurons with cyclical ITD response functions were were observed in 13 of 45 cells (28.9%; 6 DTNs and 7 non-DTNs) tested at +10 dB (re threshold), and 8 of 34 cells (23.5%; 3 DTNs and 5 non-DTNs) tested at +20 dB (re threshold). To determine if the oscillations of cyclical ITD response functions were related to the frequency of the ITD stimulus (i.e., if the interval between successive response peaks or troughs occurred at intervals related to the stimulus period; Grothe et al., 2010), we performed a fast Fourier transform on the data comprising each ITD response function. There was no correlation between the frequency of the pure tone ITD stimulus and the dominant (non-DC) frequency of the cyclical ITD response function measured at either +10 or +20 dB above threshold (data not shown). This result was unsurprising because we did not expect the spiking responses of midbrain neurons to be able to phase-lock at the mainly ultrasonic (i.e., >20 kHz) frequencies that we employed for ITD testing and to which the bat is most sensitive.

The third and final type of ITD response function we observed was from cells with monotonic (or near-monotonic) curves (note: near-monotonic also includes the stepped/peak ITD response functions recorded from the IC of *A. pallidus* and defined by Fuzessery, 1997). Neurons with monotonic ITD response functions were selective for contralateral sound sources when the function gradually increased from negative to positive values within the biologically relevant ITD range, and were selective for ipsilateral sound sources when the function gradually decreased from negative to positive values within the biologically relevant ITD range. Figure 4C shows an example of a monotonic ITD response function recorded from a shortpass DTN. This cell was selective for sounds originating from the contralateral hemisphere because its ITD function gradually increased from negative (ipsilateral ear leads) to positive (contralateral ear leads) ITD values across the biological ITD range, eventually saturating at the most extreme positive ITD values demonstrating that this cell responded best to binaural stimulation when the sound arrived first in the contralateral ear. Midbrain neurons with monotonic or near-monontic ITD response functions were observed in 8 of 45 cells (17.8%; 6 DTNs and 2 non-DTNs) tested at +10 dB (re threshold), and 11 of 34 cells (32.4%; 7 DTNs and 4 non-DTNs) tested at +20 dB (re threshold).

Table 5 provides the mean ± SE and range of peak spike counts that were evoked by the stimuli we employed during ITD testing, broken down by cell type. These results indicate there were no major differences in neural responsiveness to the signals we presented for ITD testing across the different cell populations. Table 6 summarizes the type of binaural interactions that were observed for neurons with different types of ITD response selectivity.

**Table 5:** **Range and average peak spike counts (spikes per stimulus) for cells tested with ITD stimuli at +10 dB above threshold**.

	**Cell type**	***n***	**Peak spiking response**	
			**Range**	**Mean ± *SE***
DTNs	Shortpass	16	1.10-7.40	2.44 ± 0.37
	Bandpass	5	0.73-2.50	1.61 ± 0.35
	Longpass	2	1.70-2.33	2.02 ± 0.32
Non-DTNs	Phasic	18	1.10-7.40	1.56 ± 0.23
	Sustained	4	0.60-1.70	1.36 ± 0.26

**Table 6:** **Binaural interactions of IC neurons with different types of ITD response functions**.

**Amplitude**	**ITD Response**	**EE**	**EI**	**EO**	**?**
+ 10 dB	Non-Selective	7 (2)	11 (5)	5 (3)	1 (1)
	Cyclical	4 (3)	7 (2)	2 (1)	0 (0)
	Monotonic	1 (1)	7 (5)	0 (0)	0 (0)
+ 20 dB	Non-Selective	4 (1)	5 (3)	6 (3)	0 (0)
	Cyclical	3 (1)	5 (2)	0 (0)	0 (0)
	Monotonic	2 (2)	9 (5)	0 (0)	0 (0)

Number of DTNs shown in parentheses. EE, cell excited by stimulation of contralateral and excited by stimulation of ipsilateral ear; EI, cell excited by stimulation of contralateral ear and inhibited by stimulation of ipsilateral ear; EO, cell excited by stimulation of contralateral ear and neither excited nor inhibited by stimulation of ipsilateral ear; ?, binaural inputs to cell not tested.

Table 7 compares the electrophysiological parameters of DTNs and non-DTNs with ITD-selective response functions measured at +10 and +20 dB (re threshold). For the purpose of this analysis, cells with cyclical and monotonic (or near-monotonic) ITD response functions were grouped together under a single “ITD-selective” response category for comparison with “non-ITD-selective” cells. There was no difference in the proportion of DTNs and non-DTNs that exhibited ITD selectivity at +10 dB (Fisher's exact test, *p* = 0.5544) or +20 dB re threshold (Fisher's exact test, *p* = 1.0000). We also compared the mean ± SD electrophysiological response characteristics of DTNs and non-DTNs, but only for those cells with ITD selectivity at either +10 or +20 dB (re threshold), and found no differences in the dorsal-ventral recording electrode depth, BEF, or acoustic threshold. This result indicates that there were no overt topographical differences between DTNs and non-DTNs tested with ITD stimuli in our dataset. At +10 dB (re threshold) there was a trend suggesting that DTNs were tested with shorter duration tones during ITD testing, and at +20 dB (re threshold) this difference was significant revealing that DTNs with ITD selectivity were tested with significantly shorter duration signals than non-DTNs with ITD selectivity. At both levels above threshold there was also a trend for DTNs with ITD selectivity to have higher BEFs than non-DTNs with ITD selectivity (Table 7). It is also evident from the data in Table 7 that the mean ITD_50_ value for DTNs with monotonic (or near-monotonic) ITD response functions was located outside the biological ITD range for *Eptesicus fuscus*, whereas this was not true for the population of non-DTNs. Nevertheless, given our small sample size there was no difference in the ITD_50_ values or slope factors between DTNs and non-DTNs. The fitted four-parameter sigmoid curve for one of the four non-DTNs at +20 dB (re threshold) yielded an extremely large (i.e., sharp) slope factor that we considered to be an outlier and therefore removed from the population average in Table 7; however, even when this data point was included (raw values = 599.643, −2.116, 9.965, 4.007), there was still no significant difference in the ITD slope factor between DTNs and non-DTNs (*t*-test, *p* = 0.2030).

**Table 7:** **Comparison of electrophysiological parameters of DTNs and non-DTNs with ITD-selective response functions**.

**Amplitude**	**Parameter**	**DTN**		**Non-DTN**		***t*-test**
		**Mean ± *SD***	***n***	**Mean ± *SD***	***n***	***p*-value**
+ 10 dB	Depth (μm)	1231.42 ± 269.61	12	1291.11 ± 359.70	9	0.6839
	Duration (ms)	2.58 ± 1.32	12	5.89 ± 5.11	9	0.0552
	Frequency (kHz)	47.00 ± 11.65	12	38.22 ± 9.06	9	0.0905
	Threshold (dB SPL)	44.42 ± 13.80	12	47.22 ± 18.37	9	0.7081
	ITD_50_ (μs)	162.74 ± 444.32	6	15.63 ± 21.62	2	0.6993
	ITD Slope Factor	4.60 ± 3.76	6	4.78 ± 3.40	2	0.9598
+ 20 dB	Depth (μm)	1340.30 ± 333.98	10	1274.44 ± 341.26	9	0.6929
	Duration (ms)	1.80 ± 1.08	10	6.22 ± 5.01	9	**0.0196**
	Frequency (kHz)	52.50 ± 13.06	10	39.67 ± 12.75	9	0.0566
	Threshold (dB SPL)	46.20 ± 12.79	10	48.22 ± 18.95	9	0.7979
	ITD_50_ (μs)	142.40 ± 408.38	7	28.93 ± 96.25	4	0.6326
	ITD Slope Factor	4.80 ± 5.83	7	3.95 ± 6.04	3	0.8519

Statistically significant values are indicated in bold face type.

#### 3.2.3. Neural selectivity to ILD and ITD cues

So far our analysis has examined only isolated subsets of cells looking for ILD- or ITD-selective responses at two suprathreshold levels. In this section we explore neural sensitivity to both binaural cues. Within our dataset of 56 neurons we were able to test 43 (21 DTNs) cells for selectivity to both ILD and ITD at +10 dB (re threshold), and 34 (17 DTNs) cells at +20 dB (re threshold). The data in Table 8 reveal that a surprising number of neurons had response functions with selectivity to both binaural cues. At +10 dB (re threshold) 17 of 43 (39.5%; 10 DTNs) cells were selective to ILD and ITD, whereas at +20 dB (re threshold) 17 of 34 (50.0%; 10 DTNs) cells remained selective to both ILD and ITD cues. When the response function of a neuron was selective to only a single binaural cue, most often it was selective for interaural amplitude disparities.

**Table 8:** **Number of cells with ILD-selective and ITD-selective responses at two suprathreshold levels**.

**Level**	***N***	**Non-selective**	**ILD-selective**	**ITD-selective**	**ILD- & ITD-selective**
+10 dB	43 (21 DTNs)	10 (4)	13 (6)	3 (1)	17 (10)
+20 dB	34 (17 DTNs)	10 (4)	5 (3)	2 (0)	17 (10)

The number of DTNs is shown in parentheses.

We were also interested in determining the number of neurons that had ILD_50_ and ITD_50_ values within the biological range for *Eptesicus fuscus*. A large proportion of neurons with monotonic ILD-selective response functions had ILD_50_ values within the ±20 dB biological ILD range. At +10 dB above threshold, 16 of 18 DTNs (88.9%) and 13 of 17 non-DTNs (76.5%) had ILD_50_ values within the biological ILD range. At +20 dB above threshold, 16 of 18 DTNs (88.9%) and 8 of 10 non-DTNs (80.0%) had ILD_50_ values within the biological ILD range. In our dataset, neurons with monotonic ITD-selective neurons were less likely to have their ITD_50_ values located within the ±50 μs biological ITD range. At +10 dB above threshold, 1 of 6 DTNs (16.7%), and 2 of 2 non-DTNs (100.0%) had ITD_50_ values within the biological ITD range. At +20 dB above threshold, 1 of 7 DTNs (14.3%) and 1 of 4 non-DTNs (25.0%) had ITD_50_ values located within the biological ITD range.

## 4. Discussion

This study compared the response physiology of temporally-selective DTNs from the auditory midbrain with other types of midbrain neurons that were not duration-selective as a way to further our understanding of the neural mechanisms of duration selectivity and to gain insight into the possible function(s) of DTNs in normal hearing. Our results show that some DTNs from the IC of the big brown bat have responses that were selective to ILD and ITD binaural cues. Indeed, within the auditory midbrain of *Eptesicus fuscus* DTNs were as likely as non-DTNs to have binaurally-selective responses. Therefore, in addition to having selectivity to stimulus frequency, amplitude, and duration (Morrison et al., 2014), the present results demonstrate that some DTNs from the auditory midbrain of the bat also have responses selective to interaural amplitude and timing disparities. This suggests that some DTNs may play a role in the encoding of sound source spatial location. Together with previous results on the amplitude tolerance of duration tuning (Fremouw et al., 2005), the present findings suggest that the responses of DTNs could function within the CNS as level-dependent spatio-spectro-temporal auditory filters.

Previous research on the neural mechanisms underlying duration tuning has shown that DTNs arise from the temporal interplay of excitatory and inhibitory synaptic inputs that are offset in time (Casseday et al., 1994; Fuzessery and Hall, 1999; Casseday et al., 2000; Faure et al., 2003; Aubie et al., 2009, 2012). Recently, dichotic paired tone stimulation was used to measure the strength and time-course of sound-activated inhibitory inputs to DTNs in the IC of *Eptesicus fuscus* recruited by the separate monaural and binaural central auditory pathways. That study found that the monaural pathways contain all of the circuitry necessary for creating the duration-tuned response physiology (Sayegh et al., 2014). Approximately half of the DTNs tested with dichotic paired tone stimulation, with a BD tone presented to the contralateral ear and a longer duration non-excitatory tone presented to the ipsilateral ear, showed no evidence of spike suppression when the ipsilateral ear was acoustically stimulated. And for those cells with clear evidence of spike suppression in response to ipsilateral stimulation, the recruited inhibition was weaker in strength, shorter in duration, and occurred at a longer latency compared to strength and time-course of the contalateral inhibition that created the cell's duration-selective response physiology (Sayegh et al., 2014). This latter result was consistent with the present finding that 17 of 18 DTNs with monotonic ILD response functions were classified as having EI binaural inputs (Table 3). Although the exact role of ipsilateral inhibition to DTNs is still unclear, one likely possibility is that it modulates the contralaterally-evoked excitation received by the cell thus shaping its binaural amplitude selectivity and ILD response function. Indeed, a brain circuit that sums excitation from one ear with inhibition from the other ear naturally creates neural tuning for horizontal (azimuthal) positions that could be used to compute sound source location (Grothe et al., 2010).

### 4.1. ILD selectivity

The accuracy of sound localization by echolocating bats will depend on the reception of robust binaural cues that are created, in part, by the directional properties of the torso, head, and pinnae. The head-related transfer function (HRTF) describes how ILD and ITD cues are generated and received by the tympanic membrane as a function of the spatial location of a sound stimulus. And while specific to an individual, the HRTF also varies with sound frequency. For example, low frequency sounds with long wavelengths will diffract more easily around a bat's head and torso, creating small ILD cues at those frequencies. Directional properties of the bat's pinnae and tragus also influence the HRTF through frequency-dependent destructive (attenuation) and constructive (amplification) interference patterns that are generated as the received sound energy reflects off the pinnae and tragus through the external auditory meatus and into the auditory canal (Obrist et al., 1993; Fuzessery, 1996; Firzlaff and Schuller, 2003; Aytekin et al., 2004).

The range of biologically relevant ILDs in *Eptesicus fuscus* corresponds to the depth of the sound shadow between the ears and varies with sound frequency and source location (Aytekin et al., 2004). One way to measure how ILD cues vary in the bat is to place a small microphone at the location of the tympanic membrane in each ear and broadcast sounds of different frequencies from different spatial locations. Koay et al. (1998) found that for a speaker placed 30° off from the auditory midline this corresponded to an ILD of ca. 10 dB at 32 kHz. With a speaker placed 90° from the midline Jen and Chen (1988) reported an ILD of ca. 15 dB at 25 kHz and 20–25 dB for sounds ranging from 45–85 kHz. Aytekin et al. (2004) reported that the ILD for *Eptesicus fuscus* changed monotonically with source azimuth at frequencies ≤40 kHz, whereas at higher frequencies the pattern of change was more complex and varied non-linearly with changes in both azimuth and elevation and could even result in the generation of negative ILDs (i.e., higher SPLs in the contralateral ear).

In our recordings from the IC of *Eptesicus fuscus* we found that in 29 of 35 cells (82.86%) with monotonic ILD response functions tested at +10 dB (re threshold) the steepest slope of the curve fell within the biological range of ILDs available to the bat (Figure 2). Moreover, when we examined a histogram of ILD_50_ values measured from DTNs and non-DTNs with monotonic ILD response functions, the population distribution was slightly biased toward the ipsilateral hemifield and negative ILD_50_ values (Figure 3A,B), a finding consistent with electrophysiology data from the IC of the Mexican free-tailed bat, *Tadarida brasiliensis* (Park, 1998; Park et al., 2004). If the slope of the ILD response function is important for computing sound source location, then our data suggest that many neurons in the IC of *Eptesicus fuscus* are sensitive to small ILDs and have responses selective for detecting source locations positioned just ipsilateral to the auditory midline. Future studies comparing the responses of DTNs and non-DTNs are warrented and should test the ILD selectivity of these cells at higher stimulus levels above threshold.

### 4.2. ITD selectivity

Previous studies examining the ITD selectivity of central auditory neurons in echolocating bats have used larger ITD step sizes and tested neurons over a wider range of binaural timing disparities (Harnischfeger et al., 1985; Pollak, 1988; Fuzessery, 1997). In this study we opted to test the ITD selectivity of DTNs and non-DTNs with a stimulus step size of 10 μs over an ITD range spanning both the maximum biological range for the big brown bat (50 μs) and beyond, and found that some IC neurons had responses selective to ITDs that would naturally occur in *Eptesicus fuscus*. Moreover, the ITD selectivity of these neurons could be categorized in a manner similar to the ITD sensitivity of cells found in the IC of *Antrozous pallidus* (Fuzessery, 1997), a gleaning insectivorous bat that uses high frequency echolocation for orientation and obstacle avoidance and low frequency passive listening for the detecting and localization of prey-generated sounds (Fuzessery et al., 1993). Although the overall proportion of neurons with ITD-selective response functions (cyclical plus monotonic) in the IC of *Eptesicus fuscus* was similar to that reported for the IC of *A. pallidus* (Fuzessery, 1997), cyclical neurons (Figure 4B) were more common in the big brown bat. The role(s) of neurons with cyclical ITD response functions to hearing and/or echolocation by bats is unclear because their spike counts varied greatly with small changes in ITD and the intervals between successive peaks (or troughs) within the ITD response function were unrelated to the period of the ITD acoustic stimulus. Perhaps cyclical neurons play a role in discriminating sound sources separated by very small interaural timing disparities corresponding to small angles of separation (Simmons, 1973; Simmons et al., 1983). Further studies on the response properties of DTNs and non-DTNs with cyclical ITD response functions are needed to determine if the oscillations are repeatable and therefore suggestive of some function in sound localization or if they are simply random variations in the spike count and thus inconsistently related to binaural timing disparities. We also found midbrain neurons with monotonic ITD response functions (Figure 4C), and nearly half of those cells had ITD_50_ values within the biological ITD range for *Eptesicus fuscus*. This finding is consistent with the hypothesis that neurons with monotonic (or near-monotonic) ITD response functions may contribute to the process of sound localization if the steepest slope of their ITD curve passes through the biologically relevant range of ITDs (McAlpine et al., 2001; Grothe, 2003; Hancock and Delgutte, 2004; Grothe et al., 2010). It is important to note that our results do not demonstrate that echolocating bats use interaural echo timing differences to localize objects in three dimensional space; however, they do demonstrate that the central auditory system of the bat contains neurons with response properties suitable for detecting binaural ITDs, possibly endowing bats with the computational capacity to make use of interaural echo timing differences.

### 4.3. Time-intensity trade-off

Our study characterized the ILD- and ITD-selectivity of DTNs and non-DTNs in isolation. That is, when we varied the stimulus ILD, the ITD was held constant at 0 μs suggestive of a source location along the auditory midline (i.e., 0° azimuth). Similarly, when we varied the stimulus ITD, the ILD was held constant at 0 dB which was also suggestive of a sound source located somewhere along the auditory midline (i.e., 0° azimuth). When animals localize sounds in the real world, ITD and ILD cues co-vary and are concordant with one another along with other localization cues (e.g., interaural spectral differences and absolute SPL). A concordant combination of all sound localization cues might alter the response properties of directionally-selective neurons in the IC; however, the duplex theory of sound localization (Rayleigh, 1907) together with acoustical (Jen and Chen, 1988) and behavioral studies of sound localization in bats (Koay et al., 1998) all suggest that *Eptesicus fuscus* primarily relies on ILD cues for localizing the high frequency biosonar sounds that it emits during echolocation. Surprisingly, our data indicate that within the IC of *Eptesicus fuscus* there are subsets of neurons—some which are duration-selective and others that are not—with responses selective to ITD information at ultrasonic frequencies. Previous studies on neural specializations for binaural sound localization in bats have shown that ITD stimuli can modulate the responses of some neurons to ILD cues and *vice versa* (Harnischfeger et al., 1985; Pollak, 1988; Fuzessery, 1997). This feature is known as time-intensity trading. In central auditory neurons of *Molossus ater* this time-intensity trade-off ranged between 8-50 μs/dB (Harnischfeger et al., 1985), while in *Tadarida brasiliensis* and *A. pallidus* the average trade-off was 47 μs/dB (Pollak, 1988) and 18 μs/dB (Fuzessery, 1997), respectively. Such time-intensity trade-offs have led to the hypothesis that neural mechanisms underlying high-frequency ILD sensitivity in bats are based on the relative latencies of sound-activated inputs to a neuron evoked by stimulation of the excitatory (contralateral) and inhibitory (ipsilateral) ears (Fuzessery, 1997). Therefore, varying the interaural time of arrival of a stimulus directly affects this mechanism. This led to the idea that ITD selectivity by high-frequency neurons in bats was simply a by-product of the relative latency comparison underlying ILD selectivity (Pollak, 1988) rather than an integrative mechanism combining neural measures of ILD and ITD selectivity to enhance the spatial discrimination of sound sources (Harnischfeger et al., 1985). Although our study examined the ILD and ITD selectivity of midbrain DTNs and non-DTNs in isolation, we managed to test a number of cells with both types of binaural cues and found that a majority of neurons with selectivity to one cue were also selective to the other (Table 8). Therefore, it is possible that when we observed high-frequency ITD selectivity in a IC neuron it could have been a by-product of the mechanism underlying high-frequency ILD selectivity. Future studies are needed to determine the existence and magnitude of time-intensity trading in DTNs and non-DTNs from the IC of the bat.

## 5. Summary

1. Prior to this report, the responses of auditory DTNs were known to be selective to stimulus frequency, amplitude and, of course, duration. Here we show that some DTNs from the IC of the big brown bat have responses that are also selective to binaural ILD and ITD cues, suggestive of some role in sound localization. Moreover, we found that DTNs were as likely as non-DTNs to have responses that were selective for binaural ILD and ITD cues.
2. We compared the selectivity of DTNs and non-DTNs with monotonic ILD response functions and found that the distribution of ILD_50_ values and slope factors did not differ between the two populations. We also found that the steepest slope of a neuron's ILD response function fell within the biologically relevant range of ILDs for the big brown bat, suggesting that responses of these cells could serve a role in sound localization.
3. Although ITD selectivity is thought to be more important in low-frequency hearing, we found that nearly half of the IC neurons we tested in *Eptesicus fuscus* had some form of ITD selectivity. Moreover, DTNs were as likely as non-DTNs to exhibit ITD-selective response functions. It is not clear if the ITD selectivity we observed was simply a by-product of the neural mechanisms underlying ILD selectivity, or if midbrain neurons with ITD-selective response functions are used by echolocating bats to determine the spatial location of sound sources.
4. Given that both DTNs and non-DTNs from the IC of the big brown bat had responses that were selective to either ILD, ITD, or both ILD and ITD binaural cues, this suggests that these neurons may function in neural circuits that compute sound source location. In both echolocating and non-echolocating animals, including humans, DTNs could function as level-dependent, spatio-spectro-temporal auditory filters.

## Author contributions

Manuscript conceived by Riziq Sayegh and Paul A. Faure. Data collected by Riziq Sayegh and Brandon Aubie, and analyzed by Riziq Sayegh and Paul A. Faure. Manuscript written and revised by all authors.

### Conflict of interest statement

The authors declare that the research was conducted in the absence of any commercial or financial relationships that could be construed as a potential conflict of interest.
